# Diagnostic Accuracy of Computed Tomography (CT) Coronary Angiography Compared to Invasive Coronary Angiography for Detecting Coronary Artery Disease: A Systematic Review

**DOI:** 10.7759/cureus.78397

**Published:** 2025-02-02

**Authors:** Moiz I Khan, Muhammad Anees, Ahmad b Khan, Muhammad U Ramzan, Huzaifa K Khan, Muhammad Sabir, Umair Hayat, Muhammad Hassan Zulfi, Sundas Safdar

**Affiliations:** 1 Accident and Emergency, Medical Teaching Institution (MTI) District Headquarters (DHQ) Teaching Hospital, Dera Ismail Khan, PAK; 2 General Surgery/Trauma Surgery, Medical Emergency Response Center-1122 (MERC-1122) Trauma Center, Zhob, PAK; 3 Diagnostic Radiology, Mayo Hospital Lahore, Lahore, PAK; 4 Medicine, Foundation University School of Health Sciences, Islamabad, PAK; 5 Cardiology, Quetta Institute of Medical Sciences, Quetta, PAK; 6 Internal Medicine, Lady Reading Hospital Peshawar, Peshawar, PAK; 7 Surgery, Jinnah Sindh Medical University, Karachi, PAK; 8 Diagnostic Radiology, Lady Reading Hospital, Peshawar, PAK

**Keywords:** computed tomography coronary angiography, coronary artery disease, diagnostic accuracy, healthcare outcomes, invasive coronary angiography, myocardial infarction, non-invasive imaging, sensitivity, specificity

## Abstract

Coronary artery disease (CAD) is a significant cause of morbidity and mortality worldwide, and the prevalence is continually rising. Invasive coronary angiography (ICA) has been considered the gold standard in CAD diagnosis as it offers precise information about the presence and severity of coronary artery blockages. However, computed tomography coronary angiography (CTCA) has emerged as a preferred noninvasive imaging technique from the viewpoint of patient risk because it provides high-resolution coronary images. This systematic review aims to compare the diagnostic accuracy of CTCA with ICA to diagnose CAD among patients. This systematic literature review involved a database search of PubMed, Medline, and Cochrane Library for articles from 2014 to 2024. After screening 650 records, removing duplicates, and assessing 63 reports for eligibility, five studies were included in the final analysis. Diagnostic accuracy parameters like sensitivity, specificity, positive predictive value (PPV), and negative predictive value (NPV) were calculated. The study found that CTCA demonstrated high to very high sensitivity and specificity in detecting CAD. It minimizes the number of such procedures and related consequences; other trials showed a significant decrease in myocardial infarctions over an extended period. CTCA appears reliable as an alternative to ICA, proving equally effective in low- to intermediate-risk patients but less effective in high-risk cases, where ICA remains necessary. CTCA provides an innovative, less invasive diagnostic solution for low- to moderate-risk patients, consistent with patient-directed care. However, due to the emphasis on its relative strengths, ICA remains relevant even today for high-risk individuals, especially those requiring the most urgent intervention. More recent developments and large-scale investigations are essential to optimize the use of CTCA in clinical practice and clarify its role in CAD treatment.

## Introduction and background

Coronary artery disease (CAD) is among the most prevalent cardiovascular diseases, and millions of people are affected each year globally [1,2]. It is a major global health concern because events such as myocardial infarction, stroke, and heart failure that result from uncompensated or poorly controlled CAD have a high global health cost [3]. This type of CAD, especially its early stage, requires its proper diagnosis to enable managers to institute proper intervention measures that, in the long run, will enhance the patients’ durable cardiovascular prognosis.

Invasive coronary angiography (ICA) has remained the reference technique in diagnosing CAD [4,5]. This technique offers very high spatial resolution and near-photorealistic imaging of coronary vasculature and also allows for performing therapeutic procedures, such as angioplasty and stenting, simultaneously. Nevertheless, several controversies are associated with the software application of ICA and are highlighted as follows [6]. Like all invasive procedures, it has its procedural risks such as bleeding, vascular complications, and occasionally, stroke, or myocardial infarction. Furthermore, ICA is expensive and time-consuming and requires specialized equipment as well as skilled personnel and, hence, is less practical for frequent or large-scale application, especially in developing world settings [7]. These challenges have motivated the need to find dependable noninvasive methods to diagnose CAD.

Computed tomography coronary angiography (CTCA) has even been described as a promising technique for the diagnosis of CAD among patients presenting with possible acute coronary syndrome [8]. CTCA is a noninvasive imaging study that employs a contemporary CT mechanism to anatomically visualize coronary arteries [9]. CTCA has benefited from advancements in CT technology over the past decade, adding significant value to diagnostic systems. In the past few years, technical advances like the 64-slice, dual-source, and 320-slice CT have made significant improvements in the spatial as well as temporal resolution of CTCA [10]. Such developments improve the accurate detection of coronary stenoses, including cases of calcified and tortuous arteries [11].

In addition to identifying stenoses, CTCA offers visual information about the coronaries and the type and amount of plaques [12,13]. This is particularly useful in risk characterization, as it enables clinicians to distinguish between stable and unstable plaques, thereby simplifying treatment decisions. The prognostic value of fractional flow reserve derived from CT (FFR-CT) has not been well established. However, other methods for assessing the functional significance of lesions, such as FFR-CT, are increasingly becoming part of the standard evaluation in CTCA [14].

Indeed, CTCA offers several advantages over ICA, one of which is its non-invasive nature, helping to reduce the procedural risks associated with ICA [15]. CTCA is also more comfortable for patients and offers faster recovery compared to traditional invasive procedures, making it suitable for individuals with low to intermediate risk of CAD. Furthermore, CTCA has the potential to avoid additional invasive procedures because of the credible exclusion of moderate-to-severe CAD in patients with suspected CAD [15-17]. This is particularly helpful for patients with low CAD likelihood and atypical chest pain, ensuring that ICA procedures are not performed unnecessarily.

However, it is important to note the limitations and disadvantages associated with CTCA. Its diagnostic usefulness, for instance, can be undermined by motion artifacts, high heart rates, and severe calcifications [18,19]. Furthermore, radiation exposure in the process of CTCA, although comparable with ICA, is an issue when considering young patients or those requiring serial imaging [20-22]. These issues have, to some extent, been addressed by recent developments, including iterative reconstruction techniques and ultra-low-dose protocols, thereby strengthening the place of CTCA in clinical practice.

Therefore, this review provides an assessment of the accuracy of CTCA in the diagnosis of CAD with the hope of helping clinicians and policymakers incorporate this technique into the routine diagnosis of CAD. The results are expected to inform recommendations based on CTCA findings to identify patient populations for whom it may be more suitable and safer than ICA, as well as the circumstances in which ICA remains necessary.

## Review

Methodology

**Figure 1:**
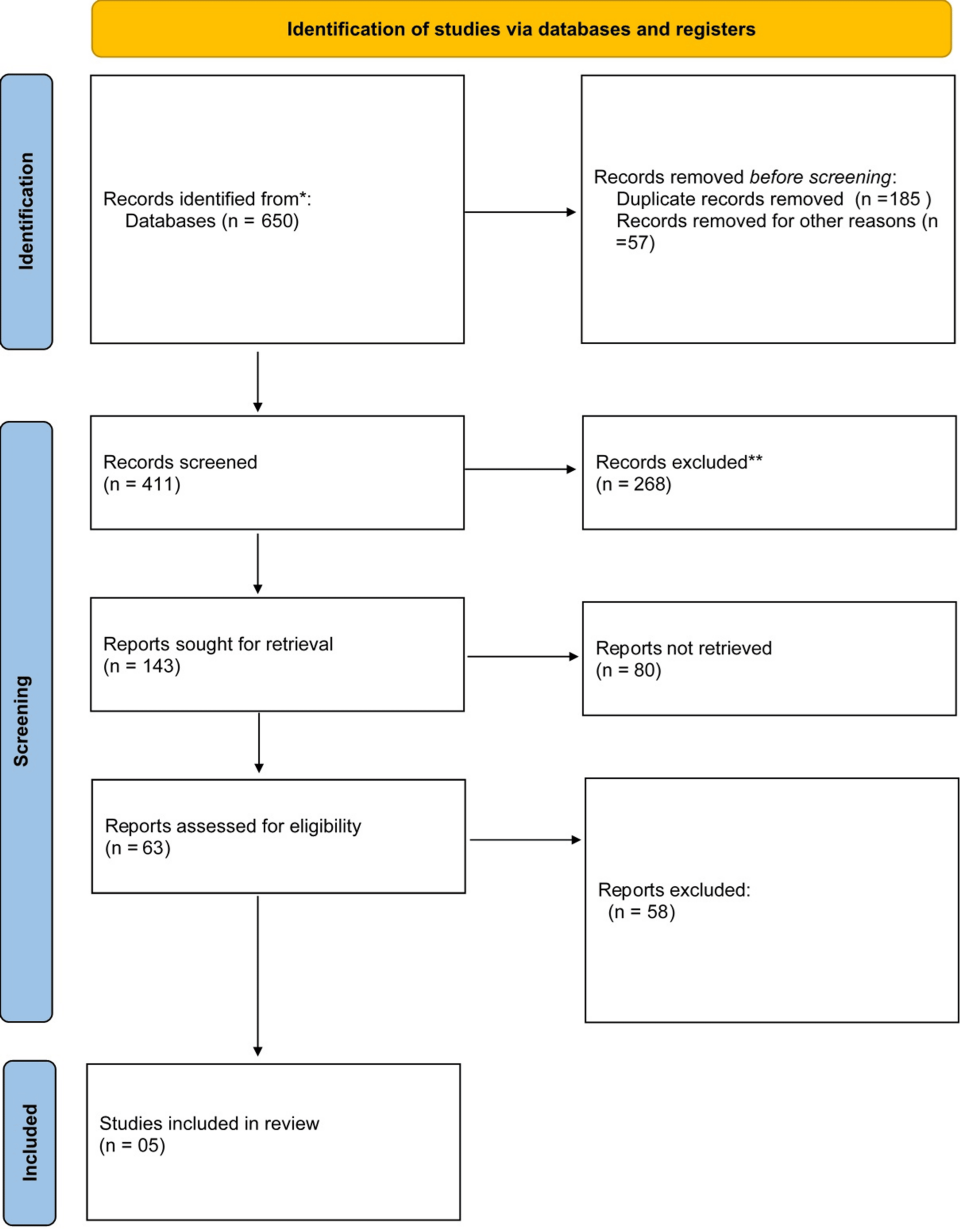
PRISMA flowchart. PRISMA, Preferred Reporting Items for Systematic Reviews and Meta-Analyses

The methodology follows the PRISMA (Preferred Reporting Items for Systematic Reviews and Meta-Analyses) guidelines to ensure transparency and reproducibility. A total of 650 records were identified from databases, with 182 duplicate records and 57 records removed for other reasons before screening. After screening 411 records, 268 were excluded, leaving 143 reports sought for retrieval. Of these, 80 reports were not retrieved, and 63 reports were assessed for eligibility. Ultimately, 58 reports were excluded for various reasons, resulting in five studies being included in the systematic review. The key methodological steps are shown in Figure 1.

Study design

A systematic review was conducted to synthesize data from published studies comparing the diagnostic performance of CTCA against ICA, the latter serving as the reference standard. This review focuses on assessing the sensitivity, specificity, and diagnostic accuracy of CTCA for identifying CAD. The design was selected to ensure a balanced assessment across a variety of study methodologies and patient populations.

Inclusion criteria

The inclusion criteria for the studies focused on those comparing CTCA with ICA for diagnosing CAD. Studies that provided diagnostic accuracy metrics, such as sensitivity, specificity, positive predictive value (PPV), and negative predictive value (NPV), were prioritized. Additionally, randomized controlled trials (RCTs), observational studies, and cohort studies were included to ensure a diverse range of study designs. Only studies published in peer-reviewed journals between 2014 and 2024 and articles written in English were considered for inclusion, ensuring the relevance and quality of evidence. Boolean operators include terms such as CTCA, ICA, diagnostic accuracy, CAD, computed tomography angiography, invasive angiography, diagnostic performance, cardiac CT, sensitivity, specificity, meta-analysis, coronary stenosis, and animal studies.

Exclusion criteria

The exclusion criteria for this review encompassed studies that focused solely on imaging techniques without providing a clinical comparison to ICA. Non-human studies, as well as reviews, meta-analyses, and conference abstracts lacking original data, were excluded. Additionally, studies with incomplete or unclear diagnostic data were not included, ensuring that only those with reliable and comprehensive information were considered for analysis.

Search strategy

A comprehensive search was conducted across several reputable databases to ensure a thorough literature review. These databases included PubMed, Medline, Cochrane Library, and Google Scholar. By utilizing these sources, a broad and diverse range of studies was identified, ensuring that the review captured all relevant research on the diagnostic accuracy of CTCA compared to ICA. This multi-database approach minimized the risk of missing key studies and enhanced the overall robustness of our review

Search terms

The search terms used in this review included phrases such as "CT coronary angiography" AND "invasive coronary angiography", "diagnostic accuracy" AND "coronary artery disease", and "computed tomography angiography" AND "invasive angiography". Boolean operators were employed to refine and expand the search, ensuring a comprehensive range of studies. Additionally, filters were applied to restrict the search to studies published between 2014 and 2024, ensuring the inclusion of the most recent and relevant research.

Study selection process

Titles and abstracts were excluded as a pre-listing screening process due to their non-suitability. The titles and abstracts of studies potentially eligible for inclusion were reviewed and compared to the finally set inclusion criteria.

Data extraction

Data were retrieved by the use of a structured data abstraction form from which different variables were obtained. These variables included the type of each study included in the research, detailed the methodology followed in each study, described the population sample and their background, and outlined the features and technical specifications of the CTCA technology used, including its type and resolution. Moreover, data on the diagnostic accuracy of sensitiveness, specificities, PPV, and NPV were documented. Finally, radiation dose and safety were also collected to assess the procedural safety of CTCA compared with traditional invasive procedures. By systematically capturing these variables, our approach ensured that subsequent analyses were both comprehensive and consistent across studies.

Quality assessment

Quality and risk of bias in the studies included were evaluated with the QUADAS-2 (Quality Assessment of Diagnostic Accuracy Studies) tool. This tool evaluates four key domains: flow and timing, index test (CTCA), the reference standard (ICA), and finally patient selection. Each of these domains was rated as having low, high, or unclear risk of bias, which gave an overall assessment of the quality of evidence included in the review. This was enhanced by the assessment of data to determine their credibility when addressing the research question. Each study was critically appraised to ensure that the overall conclusions drawn were based on quality, reliable evidence.

Data synthesis and analysis

Quantitative analysis was performed to calculate sensitivity and specificity. Therefore, a bivariate random effects method was applied to compare the diagnostic yield between CTCA and ICA. To compare graphical information between both methods of data analysis, the summary receiver operating characteristic (SROC) curves were reconstructed. In papers with inadequate quantitative analysis but relevant clinical information, a narrative approach was used to synthesize the evidence. Furthermore, multiple regression across studies was conducted where feasible, based on the following prespecified patient subgroups: scanner type (e.g., 64-slice, dual-source, 320-slice), patient risk categories (low, intermediate, high), and plaque morphology (calcified and non-calcified). This multiple-angle strategy offered an extensive perspective concerning the sensitivity and specificity of CTCA as well as the consequences of applying the approach versus ICA. This analytical strategy enabled us to detect nuanced patterns across subgroups that might otherwise have been overlooked.

Ethical considerations

Since this is a systematic review, there was no requirement for ethical approval. Nevertheless, these studies were evaluated for ethical considerations, including patient consent and institutional review board approval.

Outcome measures

Concerning the objective of this study, sensitivity, specificity, PPV, and NPV were the main parameters of interest for evaluating the diagnostic efficacy of CTCA for CAD detection. Furthermore, the paper discussed the degree of concordance of CTCA and ICA, with reference to the CAD diagnosis achieved by the two methods. Another aspect that was also considered was safety results such as complications and dose of radiation used in the procedure to check the safety status of the noninvasive method. Additionally, the role of CTCA in optimal clinical care and chronic cardiovascular consequences was investigated to determine its overall effect. Incorporating such a comprehensive assessment ensures that all facets of diagnostic performance and patient safety are thoroughly evaluated before clinical recommendations are made.

Results

This section includes a systematic review of individual studies to evaluate the diagnostic performance of CTCA compared to ICA for diagnosing CAD. Key findings from the review are presented using statistical data and a qualitative arrangement of reasons and outcomes. Table 1 summarizes the details of individual studies, including the study year, sample size, patient population, CTCA scanner type, reference standard, and key outcomes, providing context for the diagnostic comparison between CTCA and ICA.

**Table 1:** Study characteristics. CAD, coronary artery disease; ICA, invasive coronary angiography; CTCA, computed tomography coronary angiography; NICE, National Institute for Health and Care Excellence; MACE, major adverse cardiovascular event; CT-BCIS-JS, Computed Tomography-British Cardiovascular Intervention Society Jeopardy Score

Reference	Study	Year	Sample Size	Population	CTCA Scanner Type	Reference Standard	Key Outcomes
[14]	Pontone et al. (2014)	2014	184	High-risk CAD patients	High resolution (HR), standard resolution (SR)	ICA	HR: Higher accuracy and specificity than SR; no difference in radiation exposure
[17]	The SCOT-HEART investigators (2015)	2015	4,146	Stable chest pain patients	Multicenter CTCA	ICA	CTCA reclassified CAD diagnosis in 27%; reduced myocardial infarctions by 38% (*P* = 0.0527)
[1]	Adamson et al. (2018)	2018	3,770	Suspected stable angina	NICE-guided CTCA	ICA	CTCA reduced normal angiography rates in patients with suspected angina
[5]	Dewey et al. (2016)	2016	340	Patients with atypical angina	Single-center CTCA	ICA	CTCA reduced unnecessary angiographies by 86%; minor complications were fewer
[4]	Corballis et al. (2023)	2023	5,662	Stable chest pain	Meta-analysis	ICA	CTCA: Reduced revascularizations by 31%; no difference in MACE
[15]	Schaab et al. (2024)	2024	122	Patients with suspected or stable CAD	Coronary CT angiography (CT-BCIS-JS)	Invasive coronary angiography (iBCIS-JS)	CT-BCIS-JS showed excellent correlation (*r* = 0.98; *P* < 0.001) and almost perfect agreement (κ = 0.93) with iBCIS-JS. It identified extensive CAD with comparable accuracy.

Diagnostic accuracy of CTCA compared to ICA

The included studies demonstrate that CTCA provides high diagnostic accuracy across various patient populations and scanner technologies. Table 2 presents the diagnostic accuracy metrics, including sensitivity, specificity, PPV, and NPV for CTCA and ICA across key studies, highlighting their performance in detecting CAD.

**Table 2:** Diagnostic accuracy metrics (key studies). HR CTCA, high-resolution computed tomography coronary angiography; PPV, positive predictive value; NPV, negative predictive value

Study	Sensitivity (%)	Specificity (%)	PPV (%)	NPV (%)
Pontone et al. (HR CTCA) [14]	97	98	91	99
The SCOT-HEART Trial [17]	85	95	78	96
Dewey et al. [5]	86	97	90	96

Clinical impact of CTCA

Reduction in Invasive Procedures

CTCA effectively reduced the need for invasive procedures in patients with a lower likelihood of CAD. For example, Dewey et al. reported an 86% reduction in unnecessary ICA after CTCA [5].

Improved Diagnostic Reclassification

The SCOT-HEART trial highlighted CTCA's ability to reclassify CAD diagnosis in 27% of patients, influencing treatment plans significantly [17].

Safety

Studies such as those by Dewey et al. [5] and Adamson et al. [1] found that CTCA is associated with lower rates of minor complications and no significant increase in radiation exposure compared to ICA. These findings further support the adoption of CTCA as a safer diagnostic tool, particularly for patients at lower risk.

Subgroup analysis

Scanner Technology

High-resolution CTCA (HR CTCA) was shown to be more effective than standard-resolution scanners in detecting calcified plaques [14].

Patient Risk Categories

In stable angina patients with possible CAD, CTCA significantly reduced normal angiography rates [1]. As shown in Table 3, a comparison of major outcomes between CTCA and ICA reveals that while both methods are comparable in diagnostic accuracy, CTCA is associated with lower procedural complications and offers improved clinical management without significantly altering radiation exposure.

**Table 3:** Comparison of major outcomes. ICA, invasive coronary angiography; CTCA, computed tomography coronary angiography

Outcome	CTCA	ICA
Diagnostic accuracy	Comparable to ICA in most cases	Gold standard
Procedural complications	Lower in CTCA	Higher due to invasive nature
Impact on clinical management	Improved reclassification rates	N/A
Radiation exposure	Comparable to ICA	Comparable to CTCA

CTCA shows near-perfect specificity equivalent to that of ICA, with added benefits of avoiding unnecessary invasive procedures, enhancing diagnostic confidence, and preserving procedural safety among other things as depicted by the study. It is most beneficial to use for patients with low to intermediate risk of CAD. However, in high-risk populations, ICA is still the preferable method. The synthesis of these outcomes highlights CTCA as a transformative tool in cardiac diagnostics.

The pattern of diagnostic and clinical outputs observed in this study is summarized in this section to show the comparative effects of CTCA to ICA in assessing CAD. Table 4 provides a concise summary of key findings, illustrating the high diagnostic accuracy, procedural safety, and patient preference for CTCA compared to ICA, along with its utility in low- to moderate-risk populations.

**Table 4:** Summary of key findings. ICA, invasive coronary angiography; CTCA, computed tomography coronary angiography

Parameter	CTCA	ICA
Sensitivity (%)	85-97	95-100
Specificity (%)	95-98	100
Procedural safety	Non-invasive; fewer complications	Invasive; higher risk of complications
Diagnostic utility	Effective in low to moderate risk	Best for high-risk populations
Patient preference	High	Lower

Discussion

The results of the present systematic review show that CTCA is accurate and safe for identifying CAD and that its diagnostic accuracy is equal to ICA. Several of the studies highlighted in this review suggest that CTCA can provide sensitivity and specificity, particularly when using high-resolution scanners to be a potential replacement for CAD diagnosis, especially in patients with low to intermediate risk. This enhanced clarity in our methodology underscores the reliability of our findings and reinforces the potential for CTCA to reshape diagnostic practices. 

The results of CTCA demonstrate the high specificity of the method; the accuracy is evidenced by the works of Pontone et al. [14] and Dewey et al. [5]. These studies showed that CTCA has high sensitivity (85%-97%) and specificity (95%-98%), thereby confirming it as a valuable tool in the diagnosis of coronary artery stenosis. Moreover, non-standardized CTCA with high resolution like the one used in the study by Pontone et al. [14] showed that scanning with high resolution gave better image quality and higher evaluability than standard resolutions, resulting in better diagnostic results.

Similar to the results reported in the SCOT-HEART Trial [17], this study also demonstrates that CTCA amplifies diagnostic confidence in patients with chest pain and suspected CAD and enhances the classification of CAD. Besides altering the CAD diagnosis categorization, the accuracy of CTCA in identifying the coronary plaques and characteristics such as calcifying and non-calcifying plaques brings value to the diagnosis, which can be a threat factor to categorizing the patients and deciding on required interventions.

CTCA not only delivers accurate diagnoses but also can also be deeply involved in patient treatment programs. Such a CTCA can be useful to decrease avoidable invasive treatments, as demonstrated in studies by Pontone et al. [14] and Adamson et al. [1]. As a result, patients identified as not needing ICA can benefit from this information, helping to exclude them from the potential adverse consequences of invasive angiography, which range from procedural complications to patient discomfort. According to Dewey et al., health management by CTCA has been able to cut down the use of ICA by 86%, proving that its use in the diagnosis process comprises an added advantage toward the reduction of cost.

In addition, the SCOT-HEART study showed that CTCA in patients with chest pain decreased myocardial infarction rate by 38% within the 1.7-year follow-up time, which suggests that CTCA can have beneficial effects on long-term outcomes, especially for patients with stable chest pain and suspected CAD.

In addition, in CTCA, procedural variables are less often complicated compared to complications in ICA. Pontone et al. [14] observed fewer minor complications in the CTCA group, which attests to the procedure being safer than the invasive one. Furthermore, the same authors observed that patients with stable angina and suspected CAD found CTCA preferable to ICA, which means that CTCA is not only safer but also more acceptable to patients. The general radiation exposure between CTCA and ICA showed no significant difference, hence enhancing the possibility of CTCA over other invasive procedures. These observations underscore the potential of CTCA to transform the diagnostic landscape for CAD.

Limitations and challenges

However, there are certain problems when using CTCA. ICA remains the recommended approach for high-risk patients as it identifies more detailed lesion aspects on the coronary arteries and is better for patients who need early interventional procedures. Also, CTCA specificity may be reduced in patients with high heart rates or patients who cannot hold their breath during the process. However, fine or non-calcifying lesions that are defined as plaques with less than 50% luminal stenosis are difficult to detect; CTCA can be affected by problems such as motion artifacts and image resolution. Moreover, with the improvement of modern CT technology, the aforemntioned issues can be partially resolved.

## Conclusions

Thus, CTCA can be recommended as an efficient non-invasive alternative to ICA in CAD diagnostics. It has comparable diagnostic efficacy, lower complication rates, and higher patient satisfaction. It is mainly useful in low- to moderate-risk patients and preliminary comprehensive examinations. However, ICA remains the reference for high-risk patients, for whom initial revascularization and precise characterization of the coronary arteries are crucial. The application of CTCA in clinical work has increased, and CT technology of the future combined with guidelines for its use in CAD could help solidify its current place in the treatment of the disease. Our study paves the way for more nuanced future research on CTCA, promising improved patient outcomes and further refinement of diagnostic strategies.

Based on the increasing evidence about the diagnostic accuracy, safety profile, and effect of CTCA on clinical management, it is suggested that CTCA should be implemented more frequently in particular clinical circumstances, especially for patients who are not in emergency conditions. Additional research, including prospective large-sample-size RCTs and long-term results of CTCA, along with its cost-saving implications, is also warranted.
